# Effects of Gray-Scale Ultrasonography Immediate Post-Contrast on Characterization of Focal Liver Lesions

**DOI:** 10.1155/2015/193178

**Published:** 2015-05-18

**Authors:** Wei Yang, Min-Hua Chen, Wei Wu, Ying Dai, Zhi-Hui Fan

**Affiliations:** Key Laboratory of Carcinogenesis and Translational Research (Ministry of Education), Department of Ultrasound, Peking University Cancer Hospital & Institute, Haidian District, Beijing 100142, China

## Abstract

This study compared the imaging features of conventional gray scale ultrasound (US) before and after contrast-enhanced ultrasound (CEUS) for focal liver lesions and 22 evaluated the role of US post-CEUS in characterizing liver lesions. 126 patients with 158 focal liver lesions underwent CEUS and US post-CEUS examination and entered this study. There were 74 hepatocellular carcinomas (HCC), 43 hepatic metastases, and 41 hemangiomas. Imaging features of US pre-CEUS and US post-CEUS were analyzed offsite by two blinded experienced radiologists to evaluate size, boundary, echogenicity, internal texture, posterior acoustic enhancement, spatial resolution, and contrast resolution. In the end with pathological and clinical evidence, the diagnostic accuracy rate of US pre-CEUS was 53.8% (85/158 lesions), lower than that of CEUS (88.0%, 139/158 lesions); with the complementation of US post-CEUS the rate rose to 93.0% (147/158 lesions). US post-CEUS could improve the visibility of typical structures of focal liver lesions and might provide important complementary information for CEUS diagnosis. It also increases the visibility of small liver lesions compared with US pre-CEUS and helps to guide local interventional procedure.

## 1. Introduction

Clinical application of harmonic gray-scale contrast-enhanced ultrasonography (CEUS) has shown improvement in the diagnostic performance of conventional ultrasonography (US) in the characterization of focal liver lesions [1–5]. According to the dynamic perfusion characteristics during arterial, portal, and parenchymal phases which can be clearly reflected by CEUS, CEUS provided important diagnostic information and became an alternative method of enhanced CT and other imaging methods [6–8]. However, CEUS has its limitations, especially for lesions with atypical perfusion pattern or deep locations.

In the early practice with CEUS, we found that the profile and internal architecture of the liver lesions can be better displayed on conventional gray-scale ultrasound immediately after CEUS examination (US post-CEUS), compared with conventional US before CEUS (US pre-CEUS). This finding triggered the comparative study on US post-CEUS and pre-CEUS. To our knowledge, this is the first study to address this interesting phenomenon. In this study, we compared the characteristics of 158 focal liver lesions on paired US images before and after CEUS, to explore the effect of US post-CEUS on the characterization of liver lesions.

## 2. Materials and Methods

This study was approved by the Ethics Committee of Peking University Cancer Hospital, and each patient provided informed consent for the study.

### 2.1. Patients

Between October in 2011 and July in 2012, 142 patients who were diagnosed as focal liver lesions by enhanced CT, MRI, or conventional sonography were recommended for CEUS in our department and were enrolled in this study. Sixteen patients were excluded because the number of cases was too small to do statistical analysis in this study (including 3 cases of cholangiocarcinoma, 3 cases of regenerative nodule, 2 cases of focal nodular hyperplasia, and 2 cases of focal fatty sparing) or the cases had no final diagnosis (6 cases). The remaining 126 patients with 158 liver lesions formed the study group. These 126 patients underwent enhanced CT/MRI (48 cases) or biopsy (78 cases) within 1–15 days after CEUS examination. The final diagnosis was made as follows. Diagnoses of malignant lesions were confirmed by pathology (biopsy or surgery), and benign lesions were confirmed by pathology or contrast-enhanced CT or MRI with at least one-year follow-up. In patients had multiple lesions with similar appearance at CEUS image, we performed biopsy for one lesion per patient. In our group, the follow-up period was 12–17 months (median, 14 months).

The final diagnosis of the lesions showed that there were 61 patients (*n* = 50 for biopsy; *n* = 11 for resection) with 74 hepatocellular carcinomas (HCCs), 34 patients (*n* = 23 for biopsy; *n* = 11 for resection) with 43 hepatic metastatic lesions, and 31 patients (*n* = 5 for biopsy: *n* = 26 for imaging results) with 41 hemangiomas. On US pre-CEUS, the largest lesion size was 10 cm and the smallest was 0.9 cm, with the median sizes of HCC being 3.6 cm, hepatic metastasis 2.8 cm, and hemangioma 2.9 cm, respectively. Among those with HCC lesions, 52 of the 61 patients had cirrhosis and this diagnosis was made at histological and/or clinical examination. The patient group consisted of 77 males and 49 females with a median age of 54 years (age range: 20–80 years).

### 2.2. Ultrasound Instrument and Contrast Agent

Ultrasonography was performed in the Technos DU8 ultrasound system (Esaote, Italy) with real-time gray-scale contrast tuned imaging (CnTI) technique. CA430E broadband probe with frequency of 2.5 to 5.0 MHz was used. The ultrasound system GE LOGIQ 9 (Milwaukee, WI, USA) with broadband C6-1 probe (frequency: 1–6 MHz) was also used.

The contrast agent SonoVue (Bracco SpA, Milan, Italy) used in the study was supplied as a lyophilized powder, which was reconstituted by adding 5 mL of saline and gently shaking the vial by hand to form a homogenous microbubble suspension. The suspension contains 8 *μ*L/mL sulfur hexafluoride (SF6) stabilized by a phospholipid shell (microbubble concentration 5 mg/mL). The mean microbubble diameter was 2.5 *μ*m with a pH value of 4.5 to 7.5. SonoVue was administered intravenously as 2.4 mL bolus through the antecubital vein within 2-3 seconds.

### 2.3. Ultrasound Examination Method

Two sonologists performed all ultrasound examinations in this study, each with experience of at least 10 years in clinical diagnostic ultrasound and 5 years in CEUS. Each examination included obtaining three kinds of sonographic images for the same lesion using high MI conventional ultrasound before CEUS (US pre-CEUS), low MI CEUS, and high MI conventional ultrasound immediately after CEUS (US post-CEUS), respectively.

In US pre-CEUS scan, the locations, numbers, sizes, and sonographic features of the lesions were recorded and initial diagnostic result of the lesions was subjectively assessed in consensus between the two sonologists. The CEUS condition was then started and the acoustic power output was adjusted to low mechanical index (0.05–0.12) based on the lesion depth and the body habitus of the patient. After injection of contrast agent, lesions were scanned with low-acoustic-power contrast-enhanced harmonic ultrasound. The perfusion pattern and echogenicity of the lesion were observed and recorded with high definition videotape. After adequate diagnostic information of the target lesion was acquired, the whole liver was scanned quickly to detect any non-previously-seen abnormal wash-out lesion. Each CEUS scanning lasted for approximate 5-6 minutes and diagnostic result was assessed at the end of CEUS scanning. The CEUS process was followed, within 3 minutes after completion, with a high MI US post-CEUS scan by returning to baseline ultrasound status. The US post-CEUS was performed with the same scanning condition as US pre-CEUS (including plane of lesion, position of patients, ultrasound system, and probe frequency as well as the parameter of imaging), and again the features of the lesion were observed and diagnostic result was assessed.

To compare the accuracy in the evaluation and analysis of the liver tumors, ultrasound diagnoses before, during, and after contrast injection were assessed based on the ultrasound characteristics of the lesions, such as the echogenicity, morphology, texture, border, and the enhancement pattern. Each lesion was diagnosed as benign or malignant according to the established criteria (Table 1) developed based on the enhancement patterns and ultrasound features previously described [9–11].

### 2.4. Off-Site Retrospective Analysis of Grey-Scale US

Another two sonologists, each with experience of at least 10 years in clinical diagnostic ultrasound, retrospectively reviewed the US pre-CEUS and US post-CEUS imaging stored on videodisks on screen. The reading sonologists did not participate in the CEUS procedure, and they were aware of the patients' clinical histories but were blinded to the pathological results and other imaging findings. A mask placed over the screen concealed the patients' identifications. They compared the sonographic features and image quality of US post-CEUS with those of US pre-CEUS. Each lesion was assessed with eight parameters: size, margin definition, halo sign or echogenic rim, echogenicity, internal texture, posterior acoustic enhancement, spatial resolution, and contrast resolution. Halo sign, echogenic rim, and posterior acoustic enhancement were evaluated as positive or negative. Margin definition, spatial resolution, and contrast resolution were evaluated as poor, intermediate, and good. Spatial resolution was defined as the ability to differentiate two closely situated objects as distinct structures. Contrast resolution was defined as the ability to differentiate tissue structure with different echo intensity. Assessment of imaging finding was based on the consensus of the two readers.

### 2.5. Statistical Analysis

Chi-square test and Fisher test were used to compare the difference of imaging characteristics and diagnostic results. The level of significance was set at 0.05 for all tests. The statistical analysis was performed using SPSS 16.0 software (SPSS Inc., Chicago, IL).

## 3. Results

### 3.1. Diagnostic Accuracy Rate

With reference to imaging and pathological results, the diagnostic accuracy rates were shown in Table 2. They were significantly improved in CEUS compared with US pre-CEUS (*P* < 0.001). Combining CEUS with US post-CEUS findings, the diagnostic accuracy rate was further elevated 5.0% more than that of CEUS alone.

**Figure 1:**
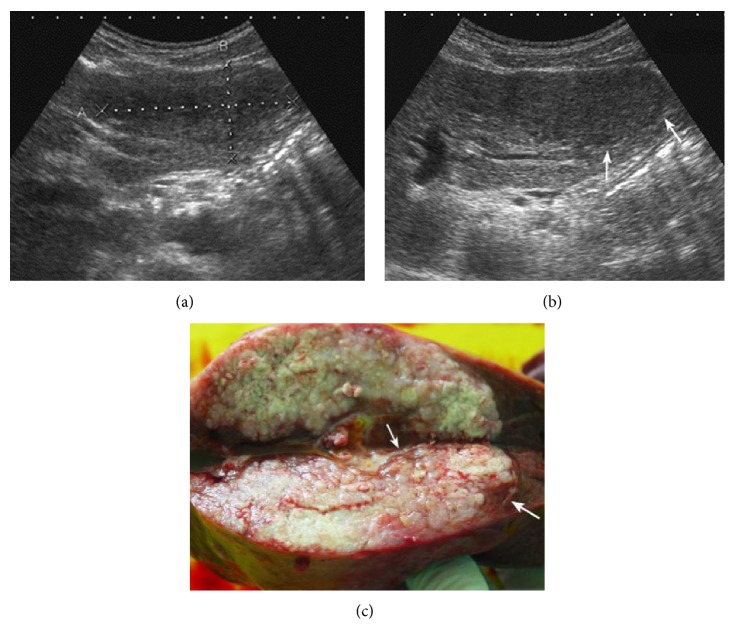
A 39-year-old man was found to have a liver tumor during the routine ultrasound examination. (a) US pre-CEUS showed an ellipsoidal tumor with heterogeneous appearance in the left liver lobe. Its front boundary was not clear due to disturbance of noise. (b) US post-CEUS showed that the tumor represented homogeneous iso-echo and had clear border and halo sign (↑) at 7 minutes after contrast agent injection. Then, the tumor was regarded as possibly malignant. (c) The tumor was confirmed as HCC by surgery pathology and gross specimen showed the fibrous membrane (↑) around the tumor, which had good correlation to halo sign on US post-CEUS.

**Figure 2:**
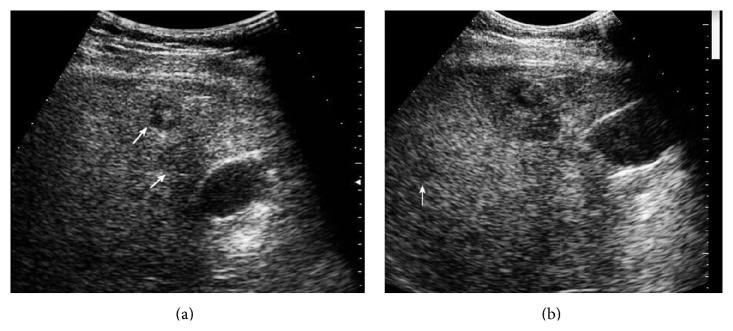
A 37-year-old woman with hepatic tumor and history of breast cancer. (a) US pre-CEUS showed multiple lesions (↑) with indefinite border in the liver. (b) US post-CEUS showed that the tumor was irregular in shape and had clear border. A new small lesion with halo sign (↑) (1.0 cm) was found in segment VIII as well at 6 minutes after contrast agent injection. This patient was considered as liver metastasis after review of US post-CUES and was confirmed by biopsy.

**Figure 3:**
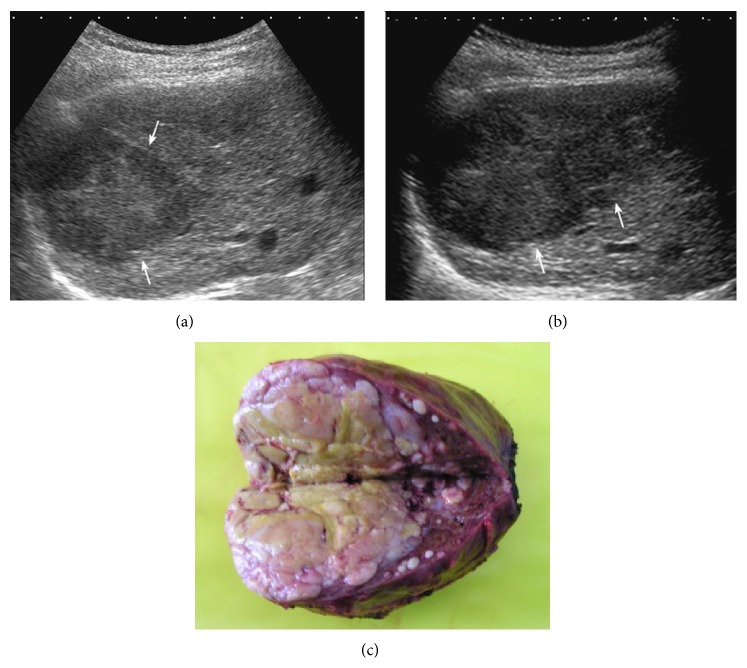
A 74-year-old man with hepatitis B for 18 years and HCC. (a) US pre-CEUS showed a large tumor with clear border in the right liver lobe. (b) US post-CEUS showed a multinodular tumor with increased size and multiple daughter lesions (↑) around the tumor at 7 minutes after contrast agent injection. (c) Surgery specimen demonstrated multinodular tumor and multiple small lesions in the adjacent area which was in accordance with US post-CEUS result.

Atypical perfusion pattern of CEUS was found in 8 HCCs, 6 hepatic metastases, and 5 hemangiomas. Post-CEUS imaging corrected the diagnostic result in 3 HCCs, 2 hepatic metastases, and 3 hemangiomas. US post-CEUS helped to clarify diagnosis in atypical CEUS cases (Figures 1, 2, and 3).

**Figure 4:**
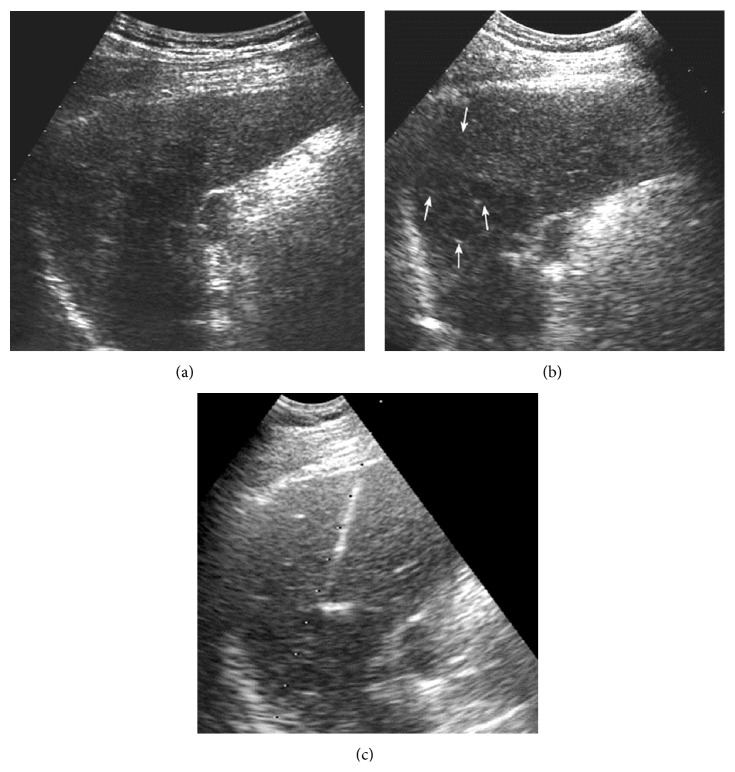
A 51-year-old man suffering from hepatitis B for 20 years with elevated AFP level of more than 400 ng/mL. (a) US pre-CEUS showed heterogeneous texture in the right liver lobe and no well-defined lesion was visible in this section. (b) US post-CEUS showed multiple small lesions with clear border (↑). (c) Biopsy was performed in these small lesions under the guidance of US post-CEUS. These new lesions were confirmed as HCC by pathology.

In addition, US post-CEUS found 28 new small lesions (<2 cm) in 18 cases, which were not detected by US pre-CEUS. Among these, 11 lesions (8 HCCs and 3 hepatic metastases) underwent biopsy or local treatment immediately under the guidance of US post-CEUS (Figure 4).

### 3.2. Comparison of Ultrasound Features between US Pre-CEUS and Post-CEUS

**Figure 5:**
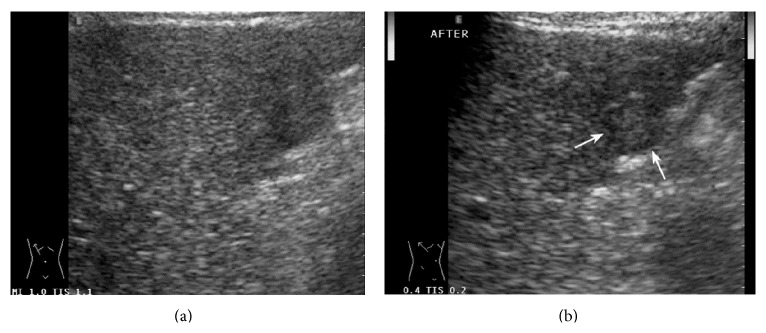
A 74-year-old man was found to have a liver tumor during the routine ultrasound examination. (a) US pre-CEUS showed an iso-echoic lesion with poor-defined margin in cirrhotic liver background. (b) US post-CEUS showed that the lesion had clearer border and a hypoechoic halo (↑) at 8 minutes after contrast agent injection. The CEUS pattern was atypical for this lesion. The diagnosis result of HCC was improved by US post-CEUS.

**Figure 6:**
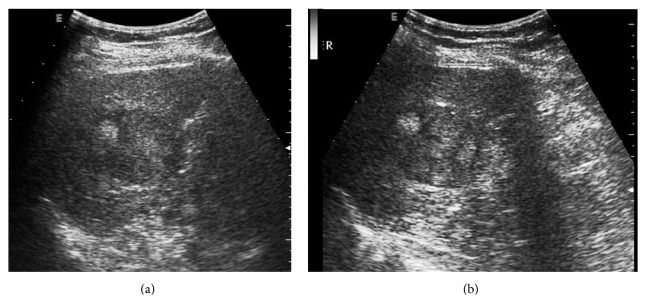
A 69-year-old woman was found to have a liver lesion. The lesion was diagnosed as benign by biopsy 2 years ago but grew up in recent days. (a) US pre-CEUS showed a tumor with clear border and heterogeneous texture in the right liver lobe. (b) US post-CEUS showed that the tumor presented clear border, halo sign, and distal sonic enhancement. The typical “nodule in nodule” and “mosaic” signs were visible in it. The lesion was regarded as HCC by post-CEUS.

*(1) HCC*. The comparative results of the imaging feature were summarized in Table 3. Compared with US pre-CEUS, US post-CEUS of the 74 HCCs examinations showed 23 lesions enlarged in sizes. US post-CEUS displayed better margin definition (*P* < 0.001), halo sign (*P* = 0.021), internal texture (*P* = 0.006), and contrast resolution (*P* < 0.001). US post-CEUS clearly showed “halo” sign in 14 more lesions and “mosaic” or “nodule in nodule” signs in 15 more lesions (Table 3; Figures 5 and 6). In HCC group, 28 lesions were smaller than 3 cm, 22 lesions were 3–5 cm, and 24 lesions were larger than 5 cm. No significant difference between them was observed with respect to tumor size.

**Figure 7:**
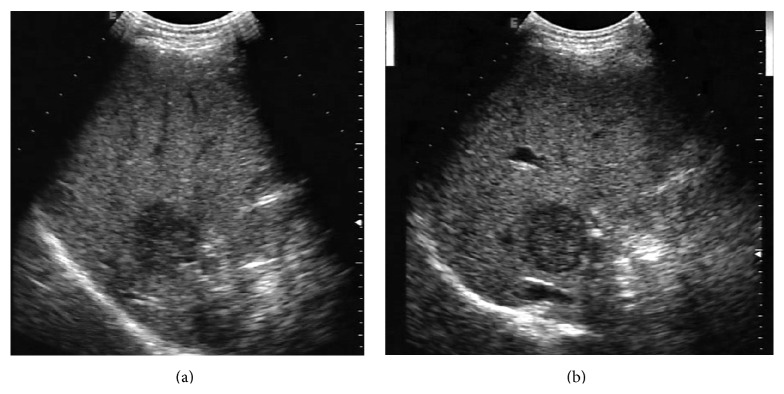
A 50-year-old man with hepatic metastases from gastric cancer. (a) US pre-CEUS showed multiple hepatic metastases in segment VIII and the largest one had irregular border. (b) US post-CEUS improved the visibility of tumor border and texture and a thin hypoechoic halo which corresponded to the peripheral enhanced zone and appeared at 6 minutes after contrast agent injection.

*(2) Hepatic Metastasis*. In 31 cases of hepatic metastasis, the primary tumors were from gastric and colorectal tract (*n* = 19), breast cancer (*n* = 6), lung cancer (*n* = 4), and other organs (*n* = 2), respectively. Of the 43 hepatic metastases, US post-CEUS showed better defined margin (*P* < 0.001) and improved contrast resolution (*P* = 0.001) (Table 4; Figure 7). In hepatic metastasis group, 25 lesions were smaller than 3 cm, 13 lesions were 3–5 cm, and 5 lesions were larger than 5 cm. No significant difference between them was observed with respect to tumor size.

**Figure 8:**
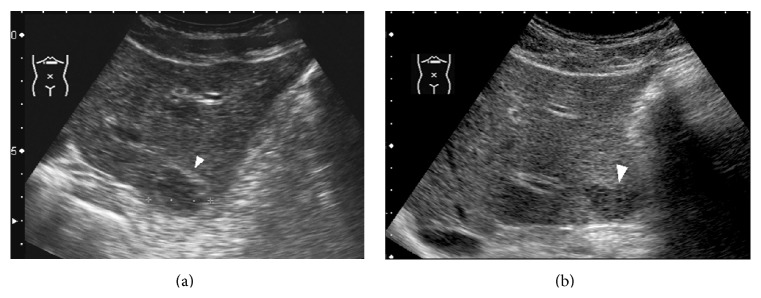
A 45-year-old woman with a hepatic hemangioma. (a) US pre-CEUS showed a hypoechoic tumor (▲) in the left liver lobe. (b) US post-CEUS clearly showed echogenicity of inner granular hypoecho (▲) at 7 minutes after contrast agent injection.

*(3) Hemangioma*. Of the 41 hemangiomas, post-CEUS showed improved visibility for margin definition (*P* = 0.043), echogenic rim (*P* = 0.003), internal texture (*P* = 0.047), and contrast resolution (*P* = 0.034) (Table 5). Post-CEUS clearly depicted echogenic rim in 13 more lesions and inner granular hypoecho in 9 more lesions (Figure 8). In hepatic metastasis group, 27 lesions were smaller than 3 cm, 8 lesions were 3–5 cm, and 6 lesions were larger than 5 cm. No significant difference between them was observed with respect to tumor size.

**Figure 9:**
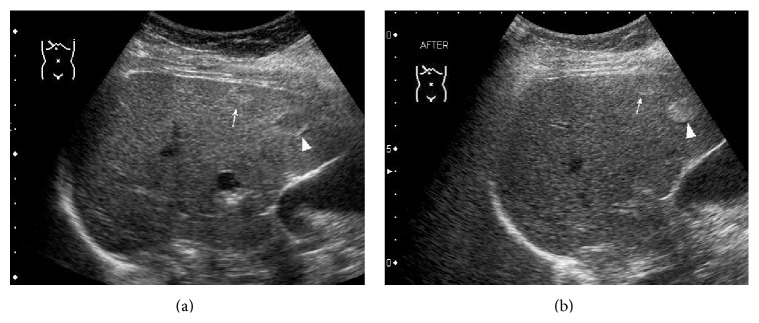
A 69-year-old woman with multiple hepatic hemangiomas. (a) US pre-CEUS showed a hyperechoic lesion (↑) near liver surface and a hypoechoic lesion (▲) near gallbladder. (b) US post-CEUS showed that the lesion (↑) near liver surface became relatively hypoechoic while the echogenicity of the lesion (▲) near gallbladder increased at 8 minutes after contrast agent injection.

The imaging results also revealed a tendency of echogenicity change between US pre-CEUS and US post-CEUS. In 21 hypoechoic hemangiomas on US pre-CEUS, echogenicity increased in 12 lesions and 9 of the 12 presented heterogeneous textures on US post-CEUS. In 14 hyperechoic hemangiomas on US pre-CEUS, echogenicity decreased in 9 lesions on US post-CEUS (Figure 9).

## 4. Discussion

The recent introduction of microbubble contrast agents and contrast-specific imaging technique opened new prospects for liver ultrasound. Several reports have shown that CEUS can substantially improve the detection and characterization of focal liver lesions with respect to baseline US studies [1–3, 12]. However, CEUS still has limitations: its spatial resolution decreased as contrast resolution increased [13]; it was more likely to be influenced by attenuation; furthermore, CEUS can only obtain the perfusion status of a relatively small interest region of the liver; thus, it has difficulty in evaluating the entire liver with one contrast injection. When the lesion is hypovascular, is located deeply, has severe cirrhotic background, or is transiently enhanced, the perfusion pattern is atypical. So it is hard to achieve definitive diagnosis (begin or malignant) by CEUS alone in these cases.

In this study, we compared the sonographic features and image quality of US post-CEUS with those of US pre-CEUS in 158 focal liver lesions and found that US post-CEUS improved the depiction rate of the lesion profile and internal pathological structures. It was found that US post-CEUS had comparative higher contrast resolution than US pre-CEUS and higher spatial resolution than CEUS. As CEUS revealed the pattern of vascular microarchitecture and the process of dynamic perfusion, US post-CEUS provided useful information on configuration and tissue structure of lesions, which could benefit accurate diagnosis.

### 4.1. Potential Mechanism for This Effect of US Post-CEUS

Ultrasound contrast media, consisting of microbubbles, are relatively larger entities (1–10 *μ*m) than X-ray or MR contrast agents. The microbubbles surviving the lung filter are confined to the vascular bed and cannot leak out to the extravascular space like X-ray or MR contrast agents [14]. SonoVue (BR1; Bracco, Milan, Italy) is a sulfur hexafluoride-filled microbubble contrast agent that is licensed for use in abdominal and vascular imaging in most European countries and China. It was the most common used contrast agent in our country. This agent has a strong nonlinear harmonic response when it is insonated with low acoustic power [15]. The lifespan of microbubbles in blood flow was comparatively longer than the first-generation CEUS agent. In clinical application with SonoVue, it is common to see a certain amount of microbubble left in liver after 5-6 minutes of low MI CEUS scanning.

A microbubble, if driven by intense ultrasound, will suffer irreversible disruption. And when the microbubble disappears as an acoustic scatter (not instantly, but over a period of time determined by the bubble composition), it emits a strong, brief nonlinear echo [16, 17]. In general, high MI was applied in non-contrast conventional US while low MI was applied in CEUS. When we finished CEUS scanning and immediately returned to conventional grey-scale US status, the remaining microbubbles in the liver would be destroyed by intense ultrasound energy and emitted strong harmonic signal. Ultrasound instruments could receive the echo signal not only from tissue but also from microbubbles exploding in this process. The remaining microbubbles accentuate the contrast between different tissue compositions and may emphasize the subtle structure of lesion on sonogram, thus benefiting lesion characterization.

### 4.2. The Role of US Post-CEUS in Improving Diagnostic Rate

#### 4.2.1. HCC and Hepatic Metastasis

Although SonoVue does not have any late liver parenchymal uptake, post-CEUS scans performed at 5-6 minutes after bolus administration generally show a persistent detectable sinusoidal enhancement of liver parenchyma, while the normal sinusoidal architecture in malignant lesion has been destroyed with manifesting as focal hypoechoic well-demarcated lesions. Well-defined margin made accurate measurement of tumor size easy, which might explain the size of tumor on US post-CEUS, especially in infiltrative HCC, and sometimes become larger than that on US pre-CEUS. A halo around a liver mass on sonogram has been regarded as a malignancy sign [18]. The hypoechoic halo observed on sonogram represents tissue with composition and acoustic impedance differing from those of tissue at the centre of and surrounding liver [19, 20]. Thus the remaining microbubbles permitted us to easily observe the hypoechoic halo on US post-CEUS scan. HCC with a “nodule in nodule” appearance is considered to be in the transitional stage from early HCC or premalignant lesion to advanced HCC [21]. Advanced HCC with central necrosis may also have heterogeneous texture like “mosaic” sign. Specific “mosaic” or “nodule in nodule” sign was easier to be detected on US post-CEUS than US pre-CEUS because the remaining microbubbles in blood pool enhanced the contrast of different histological structures or differentiated component of tumor immediately after CEUS.

In our group, 3 of 8 HCCs which were not definitively diagnosed by reading CEUS scan due to atypical perfusion pattern or obvious attenuation of liver were considered as HCC after review of US post-CEUS scan. The tumor border, halo sign, and heterogeneous appearance were better displayed on US post-CEUS, which helped us get accurate diagnosis. Of them, a 4.4 cm hyperechoic HCC was regarded as right adrenal gland tumor at first due to close relationship with right adrenal gland. CEUS did not deny this diagnosis. The tumor was later diagnosed as possible HCC by US post-CEUS because typical halo sign and “nodule in nodule” sign appeared on US post-CEUS.

In the analysis of the conventional US scanning before and after CEUS in 43 hepatic metastases, we found improvement of imaging characteristics including margin definition and contrast resolution. It should be noted that the echogenicity on US post-CEUS was related to the enhanced patterns on CEUS in some cases. The rim-like enhancement of hepatic metastasis in CEUS process reflects fibrosis or necrosis in the center of the lesion [22], which might explain the phenomenon why the echogenicity decreased on US post-CEUS in such tumors. The nodular enhancement in CEUS process reflects rich blood flow and vascular structure in the whole lesion and usually results in brighter and heterogeneous echogenicity of lesions on US post-CEUS.

In this study, US post-CEUS found more small lesions (<2 cm) in 18 cases, which were not detected by US pre-CEUS. Among them, 11 lesions (8 HCCs and 3 hepatic metastases) underwent biopsy or local treatment immediately. This proved the clinical value of US post-CEUS in detecting small lesions and guiding biopsy or local treatment of the lesion.

#### 4.2.2. Hemangioma

Hemangioma is the most common benign focal lesion in the liver and usually represents a homogeneous hyperechoic nodule. In 1993, Moody and Wilson [23] described a pattern that is strongly suggestive of hemangioma—the presence of a circumferential echogenic rim contiguous to normal liver and inner granular hypoecho. In this study, we regarded both of the US appearances as diagnostic criteria for hemangioma. The main advantage of US post-CEUS lied in the improvement of visibility of lesion border and inner architecture; as seen, that US post-CEUS revealed echogenic rim in 31 lesions and inner granular hypoecho in 18 lesions while US pre-CEUS detected these in only 18 and 9 lesions, respectively. This information helped us to accurately diagnose hemangioma. The mechanism might also be related to the fact that the remaining microbubbles in the liver after CEUS increased the contrast between hemangioma and adjacent liver parenchyma and between different tissue compositions of hemangioma.

Sometimes it is difficult to distinguish hemangioma from other liver lesions with CEUS, especially in patients with malignancy history. In one case of the study, a small hemangioma in the left liver lobe coexisted with a large HCC in the right lobe. The hemangioma was misdiagnosed as malignant based on the clinical data and its relatively quick wash-out on CEUS. After review of the US post-CEUS image, obvious echogenic rim was found around the lesion and then hemangioma was considered. Another two cases after colon-rectal carcinoma surgery had a hemangioma. The hemangioma presented atypical perfusion on CEUS but finally got accurate diagnosis with reference to the echogenic rim and inner granular hypoecho on US post-CEUS scans.

It should be noted that there was a specific “echo change” phenomenon in hemangioma on US pre- and post-CEUS. In hyperechoic hemangiomas on US pre-CEUS, echogenicity tended to decrease on US post-CEUS; in hypoechoic hemangiomas on US pre-CEUS, echogenicity tended to increase on US post-CEUS. This phenomenon might be related to the different proportion of vascular channels and fibrous septa of different hemangiomas.

### 4.3. Limitations

The US post-CEUS is a compensatory and dependent examination. It cannot bring dramatic improvement for CEUS, since CEUS already had high diagnostic accuracy rate. Furthermore, the US post-CEUS examinations should be performed within a short time after CEUS, and evaluation of US post-CEUS requires experience in scanning and observation skills. However, without injection of additional contrast agent, US post-CEUS provided important information about ultrasound features and changed diagnostic result in some cases with atypical CEUS perfusion pattern. It would be helpful and easy to do in clinical practice.

## 5. Conclusions

US post-CEUS optimized the display of tumor boundary and internal structures compared with conventional grey-scale US, thus providing additional information about pathological characteristic of liver lesions. US post-CEUS was a complementary diagnostic method for lesions with atypical CEUS pattern, and it could also guide immediate biopsy and local treatment if necessary for small lesions, which are difficult to be displayed on conventional US.

## Figures and Tables

**Table 1:** Diagnostic criteria of focal liver lesions on CEUS features.

Lesion type	CEUS features
Hepatocellular carcinoma	Diffuse enhancement at arterial phase and wash-out at portal venous or late phase

Liver metastases	Peripheral rim with viable intralesional enhancement at arterial phase and wash-out during portal venous or late phase

Hemangioma	Peripheral nodular or rim enhancement at arterial phase with centripetal progression in portal venous and late phase

**Table 2:** Diagnostic accuracy rates in different liver lesions (%).

Diseases	US pre-CEUS	CEUS	US post-CEUS
Hepatocellular carcinoma	51.4 (38/74)	89.2 (66/74)	93.2 (69/74)
Hepatic metastasis	58.1 (25/43)	86.0 (37/43)	90.7 (39/43)
Hemangioma	53.7 (22/41)	87.8 (36/41)	95.1 (39/41)

Total	53.8 (85/158)	88.0 (139/158)	93.0 (147/158)

Note: the data in parentheses refers to lesion number.

**Table 3:** Imaging characteristic of US pre-CEUS versus US post-CEUS in 74 hepatocellular carcinomas.

Imaging characteristic	Grading	Pre-CEUS	Post-CEUS	*P* value
Size	Enlarged	—	23	—

Margin definition	Poor	28 (37.8)	6 (8.1)	<0.001
	Intermediate	22 (29.8)	14 (18.9)	
	Good	24 (32.4)	54 (73.0)	

“Halo” sign	−	42 (56.8)	28 (37.8)	0.021
	+	32 (43.2)	46 (62.2)	

Echogenic rim	−	71 (95.9)	72 (97.3)	>0.05
	+	3 (4.1)	2 (2.7)	

Echogenicity	Hyperechoic	23 (31.1)	16 (21.6)	>0.05
	Iso-echoic	13 (17.6)	14 (18.9)	
	Hypoechoic	38 (51.3)	44 (59.5)	

Internal texture	Homogenous	35 (47.3)	18 (24.3)	0.004
	Heterogeneous	39 (52.7)	56 (75.7)	
	“Mosaic” or “nodule in nodule”	19	34	0.001
	Inner granular hypoecho^#^	2	3	>0.05

Posterior acoustic enhancement^*^	−	31 (56.4)	22 (40.0)	>0.05
	+	24 (43.6)	33 (60.0)	

Spatial resolution	Poor	12 (16.2)	9 (12.2)	>0.05
	Intermediate	21 (28.4)	19 (25.7)	
	Good	41 (55.4)	46 (62.1)	

Contrast resolution	Poor	28 (37.8)	10 (13.5)	<0.001
	Intermediate	26 (35.1)	18 (24.3)	
	Good	20 (27.1)	46 (62.2)	

Note: the data refers to lesion number if not specified. ^*^Sometimes, it was not feasible to observe the posterior echo of lesions due to undesirable locations. The data in parentheses were percentages.

^#^The presence of multiple small hypoechoic areas in the hyperechoic lesion.

**Table 4:** Imaging characteristic of US pre-CEUS versus US post-CEUS in 43 hepatic metastases.

Imaging characteristic	Grading	Pre-CEUS	Post-CEUS	*P* value
Size	Enlarged	—	4	—

Margin definition	Poor	14 (32.6)	3 (7.0)	<0.001
	Intermediate	18 (41.9)	11 (25.6)	
	Good	11 (25.5)	29 (67.4)	

“Halo” sign	−	24 (55.8)	18 (41.9)	>0.05
	+	19 (44.2)	25 (58.1)	

Echogenic rim	−	41 (95.3)	40 (93.0)	>0.05
	+	2 (4.7)	3 (7.0)	

Echogenicity	Hyperechoic	4 (9.3)	4 (9.3)	>0.05
	Iso-echoic	16 (37.2)	13 (30.2)	
	Hypoechoic	23 (53.5)	26 (60.5)	

Internal texture	Homogenous	24 (55.8)	20 (46.5)	>0.05
	Heterogeneous	19 (44.2)	23 (53.5)	
	“Mosaic” or “nodule in nodule”	0	2	>0.05
	Inner granular hypoecho^#^	2	5	>0.05

Posterior acoustic enhancement^*^	−	21 (58.3)	19 (52.8)	>0.05
	+	15 (41.7)	17 (47.2)	

Spatial resolution	Poor	8 (18.6)	6 (12.0)	>0.05
	Intermediate	13 (30.2)	14 (32.5)	
	Good	22 (51.2)	23 (53.5)	

Contrast resolution	Poor	11 (25.6)	3 (7.0)	0.001
	Intermediate	17 (39.5)	8 (18.6)	
	Good	15 (34.9)	32 (74.4)	

Note: the data refers to lesion number if not specified. The data in parentheses were percentages. ^*^Sometimes, it was not feasible to observe the posterior echo of lesions due to undesirable locations.

^#^The presence of multiple small hypoechoic areas in the hyperechoic lesion.

**Table 5:** Imaging characteristic of US pre-CEUS versus US post-CEUS in 41 hemangiomas.

Imaging characteristic	Grading	Pre-CEUS	Post-CEUS	*P* value
Size	Enlarged	—	6	—

Margin definition	Poor	4 (9.8)	2 (4.9)	0.043
	Intermediate	17 (41.4)	8 (19.5)	
	Good	20 (48.8)	31 (75.6)	

“Halo” sign	−	39 (95.1)	40 (97.6)	>0.05
	+	2 (4.9)	1 (2.4)	

Echogenic rim	−	23 (56.1)	10 (24.4)	0.003
	+	18 (43.9)	31 (75.6)	

Echogenicity	Hyperechoic	14 (34.2)	10 (24.4)	>0.05
	Iso-echoic	6 (14.6)	12 (29.3)	
	Hypoechoic	21 (51.2)	19 (46.3)	

Internal texture	Homogenous	24 (58.5)	15 (36.6)	0.047
	Heterogeneous	17 (41.5)	26 (63.4)	
	“Mosaic” or “nodule in nodule”	3	2	>0.05
	Inner granular hypoecho^#^	9	18	0.034

Posterior acoustic enhancement^*^	−	17 (63.0)	21 (77.8)	>0.05
	+	10 (37.0)	6 (22.2)	

Spatial resolution	Poor	8 (19.5)	6 (14.6)	>0.05
	Intermediate	13 (31.7)	11 (26.8)	
	Good	20 (48.8)	24 (58.6)	

Contrast resolution	Poor	14 (34.1)	7 (17.1)	0.034
	Intermediate	17 (41.5)	13 (31.7)	
	Good	10 (24.4)	21 (51.2)	

Note: the data refers to lesion number if not specified. The data in parentheses were percentages. ^*^Sometimes, it was not feasible to observe the posterior echo of lesions due to undesirable locations.

^#^The presence of multiple small hypoechoic areas in the hyperechoic lesion.
